# Evaluation of Bleb Morphology and Reduction in IOP and Glaucoma Medication following Implantation of a Novel Gel Stent

**DOI:** 10.1155/2017/9364910

**Published:** 2017-06-20

**Authors:** Antonio Maria Fea, Roberta Spinetta, Paola Maria Loredana Cannizzo, Giulia Consolandi, Carlo Lavia, Vittoria Aragno, Francesco Germinetti, Teresa Rolle

**Affiliations:** Clinica Oculistica, Dipartimento di Scienze Chirugiche, Universita' di Torino, Via Juvarra 19, 10100 Torino, Italy

## Abstract

**Objective:**

To evaluate the efficacy and safety of the Xen Gel Stent and provide a macro- and microscopic analyses of bleb morphology.

**Methods:**

A prospective 12-month study on patients with primary open-angle glaucoma. Patients underwent implantation of the XEN Gel Stent (Allergan INC, Dublin, Ireland) either alone or combined with a cataract surgery. Biomicroscopy, in vivo confocal microscopy (IVCM), and anterior segment-optical coherence tomography (AS-OCT) were used to assess bleb morphology. Safety parameters were adverse events, best corrected visual acuity, visual field, and corneal endothelial cell loss. A postoperative IOP ≤ 18 mmHg without or on medications was respectively defined as complete and qualified success while an IOP ≥ 18 mmHg was defined as failure.

**Results:**

Twelve eyes of 11 patients were evaluated. At one year, 5 out of 10 patients available achieved a complete success while five were qualified success. AS-OCT showed that bleb wall reflectivity was significantly higher in the failure group; IVCM revealed that stromal density was significantly lower in the success group. No safety issues were recorded.

**Conclusion:**

Implantation of the XEN Gel Stent appears to be a safe and effective procedure. AS-OCT and IVCM may be helpful in bleb assessment.

## 1. Introduction

Since the mid-1960s, trabeculectomy has been the gold standard in the treatment of glaucoma [1]. A plethora of published studies highlight the efficacy of trabeculectomy, including a 20-year follow-up study of trabeculectomy in 234 patients by Landers et al., who found that trabeculectomy survival was approximately 60% with no topical medication and approximately 90% with topical medication [2]. The procedure does, however, have limitations.

Findings from the National Survey of Trabeculectomy which included 1240 cases showed that early complications were reported in 46.6% of cases and late complications in 42.3% cases. The most frequent early complications were hyphema (24.6%), shallow anterior chamber (23.9%), hypotony (24.3%), wound leak (17.8%), and choroidal detachment (14.1%). The most frequent late complications were cataract (20.2%), visual loss (18.8%), and encapsulated bleb (3.4%) [3].

In an attempt to mitigate the intra- and postoperative complications associated with traditional glaucoma surgery, several new minimally invasive glaucoma surgeries have been developed. These include ab interno canaloplasty (ABiC™, Ellex Medical Lasers Ltd, Adelaide, Australia, the Trabectome® (NeoMedix Inc., Tustin, California), the iStent® (Glaukos, Laguna Hills, California) the Hydrus™ (Ivantis, Irvine, California), and the CyPass Suprachoroidal Microstent (Transcend Medical, Menlo Park, California) [4, 5].

Another minimally invasive device known as the XEN Gel Stent (Allergan INC, Dublin, Ireland) is a long flexible tube made of chemically treated, cross-linked gelatin; one end of which is placed in the anterior chamber allowing fluid to exit the eye and form a subconjunctival bleb at the other end of the tube [6]. Aqueous outflow is determined by the bore (45 *μ*m) and length (6.0 mm) of the stent; these particular dimensions were selected because they represent the best ratio for optimal outflow and a low rate of complications. Traditional ab externo trabeculectomy requires an incision in both the conjunctiva and sclera which may cause bleeding and scarring, potentially leading to a bleb failure. In contrast, the XEN Gel Stent is implanted using an ab interno approach. Thus, the device is designed to have similar efficacy as invasive gold standard procedures, but has the potential to significantly lower traditional complication rates [7]. Findings from a prospective, nonrandomized, multicenter evaluation of multiple XEN Gel Stent models showed that there was a 40% reduction in IOP at 36 months and a 74% reduction in antiglaucomatous medications. Moreover, the outcomes of stand-alone implants and implants combined with cataract surgery were similar [8].

One of the keys to successful filtering surgery is the development of a bleb in the postoperative period. Morphologic changes to the developing filtering bleb after surgery may help to predict early treatment failure and guide bleb revision and management [9]. An early study by Addicks et al., for example, found that failed trabeculectomy blebs had dense collagenous connective tissue in their walls, while in functioning blebs, the subepithelial connective tissue was loosely arranged and contained histologically clear spaces [10].

Currently, there is no data describing bleb morphology following implantation of the XEN Gel Stent. Consequently, we undertook a 12-month prospective study to evaluate efficacy, safety, and bleb morphology following implantation of the device, either alone or combined with cataract surgery, in patients with POAG.

## 2. Methods

### 2.1. Study Design and Patients

This was a single-center, prospective study undertaken at the Eye Clinic of the Ophthalmic Hospital in Turin, Italy. Patients were fully informed of the purpose of the study, surgical technique, potential risks, and possible outcomes. The study received approval from the local Ethics Board Committee, and written informed consent was obtained before the study procedure and data collection according to the principles specified in the Helsinki protocol and its amendments.

Patients eligible for inclusion in the study were those with a diagnosis of POAG with uncontrolled IOP (defined as ≥18 mmHg and ≤33 mmHg in between one and four antiglaucoma medications) and a healthy and mobile superior bulbar conjunctiva. Additionally, all included patients had the stent protruding at least 1 mm and no more than 2 mm in the anterior chamber as assessed by gonioscopy and at least 3 mm of the stent measurable out of the scleral track. Patients were excluded from the study if they had undergone previous corneal and glaucoma surgeries, had a diagnosis of glaucoma other than POAG, corneal opacities, or ocular disease other than glaucoma.

Postoperative surgery evaluation was performed basing on IOP and use of antiglaucoma medications; patients achieving a postoperative IOP of <18 mmHg without antiglaucoma medications were defined “complete success” and were part of the success group. The failure group was composed by patients with a postoperative IOP ≤ 18 mmHg on antiglaucoma medications, namely “qualified success,” and by those with a postoperative IOP ≥ 18 mmHg.

### 2.2. Endpoints/Outcome Measures

The primary objective of this study was to investigate bleb morphology through the follow-up and to find possible prognostic factors of surgical success.

The secondary objective was to assess the safety and efficacy of XEN Gel Stent implantation. Efficacy was assessed by reductions in IOP and medication use following implantation of the device. Safety was determined by the incidence of adverse events, loss of best corrected visual acuity (BCVA), Humphrey visual field parameters, and endothelial cell count.

### 2.3. Pre- and Postoperative Evaluations

All patients underwent a detailed clinical assessment before and after implantation of the gel stent. Preoperatively, patients underwent a slit lamp examination, gonioscopy (to classify the iridocorneal angle according to Shaffer grading scale), pachymetry, and tonometry. A full medical history, including medication use, was recorded.

Conjunctiva/bleb evaluation with biomicroscopy was performed at 1 week and at 1, 3, 6, and 12 months postoperatively. Specular microscopy (Konan Cell Check XL; Konan Medical, Irvine, CA, USA) was performed preoperatively and at 1, 3, 6, and 12 months postoperatively. CD (cell density), SD (standard deviation), CV (coefficient of variation), and 6A (hexagonality coefficient) were evaluated. Humphrey 24-2 SITA-standard visual field testing (Carl Zeiss Meditec, Dublin, CA, USA) was undertaken preoperatively and at 6 and 12 months after surgery. In vivo confocal microscopy (IVCM) was performed preoperatively and at 3, 6, and 12 months postoperative, while AS-OCT was undertaken at 1, 3, 6, and 12 months postsurgery.

Need for further intervention (e.g., needling or trabeculectomy) was based on failure in IOP control.

### 2.4. Assessment of Bleb Morphology

#### 2.4.1. Biomicroscopy

Biomicroscopy was used to measure bleb area in clock hours (1–12), vascularization (0–4 according to a modified and Moorfields Bleb Grading [11]), and borders (diffuse/well defined). Two clinicians independently evaluated the bleb. If the difference in terms of evaluation was within one, a mean was performed; if the difference was higher, the bleb was reevaluated by a third operator and a mean was taken.

#### 2.4.2. IVCM

IVCM was performed with the HRT II/Rostock Cornea Module (RCM; Heidelberg Engineering, Inc, Franklin MA, USA). Before the IVCM examination, one drop of Novesine 0.4% (oxybuprocaine 0.4%) was instilled into the lower conjunctival fornix. The patient was then seated in front of the IVCM with the head set steady in a headrest. The eye was properly aligned to obtain tangential optical section of the whole upper bulbar conjunctiva by means of a dedicated target mobile bright red light of the instrument that the patient fixed with the contralateral eye. A digital camera provided a lateral view of the eye and the position of the objective lens on the surface of the eye for each scan. Manual frame acquisition throughout the upper bulbar conjunctiva at approximately 7 mm from the superior limbus in down gaze was performed. The superonasal conjunctiva was examined at all postoperative visits; Results were reported as the average of three best images selected by the IVCM operator. The main outcome measures of IVCM were mean intraepithelial microcyst area, mean intraepithelial microcyst density, and subepithelial connective tissue density in terms of reflectivity (grayscale).

#### 2.4.3. AS-OCT

AS-OCT was performed using the RTVue-100 (Optovue, Inc, Fremont, CA, USA). For the examination, the patient was asked to gaze downwards and the upper eyelid was gently elevated, exposing the bleb as much as possible. AS-OCT longitudinal scans were used to check the appropriate placement of the device and properly orient the cross-scans, which in turn were used to perform a macroscopic analysis of bleb morphology. Three cross-scans were performed at final portion of the gel stent, and the average was considered in the calculations.

Variables of bleb morphology assessed by AS-OCT were stromal density of the bleb wall (bleb wall reflectivity, grayscale), bleb wall thickness at the thinnest point, maximal height of the bleb, and total area of the hyporeflective cystic spaces. The best image obtained in a series of three images was analyzed.

IVCM measurements of the mean intraepithelial microcyst area and the subepithelial connective tissue density and AS-OCT measurements of the total area of the hyporeflective cystic spaces and of the bleb wall reflectivity were calculated using ImageJ software (http://rsb.info.nih.gov/ij/download.html). The density of the microcysts showed by IVCM was evaluated using the manual cell counting procedure provided in the HRT software. The bleb wall thickness and the maximal height of the bleb were assessed using the AS-OCT software.

Both the IVCM and AS-OCT operators were masked for all clinically relevant data.

### 2.5. Study Device

The XEN Gel Stent is indicated for the reduction of IOP in patients with POAG where previous medical treatments have failed. The device is 6 mm long and 45 *μ*m wide and is injected through a small self-sealing corneal incision using a simple, preloaded injector. The XEN Gel Stent is made of a permanent, soft, collagen-derived gelatin. Upon implantation, it allows diffuse outflow of aqueous from the anterior chamber into the nondissected tissue of the subconjunctival space [7].

### 2.6. Surgical Procedure

All surgeries were performed under peribulbar anaesthesia. Thirty minutes before surgery, 0.02% mitomycin C (MMC) was injected into the subconjunctival space in the quadrant selected for implantation of the device. The conjunctiva in the superonasal quadrant was marked 3 mm from the limbus as a guide for the exit point of the device. A 1.5 mm inferotemporal corneal incision was then made 1 mm anterior to the limbus. Following injection of cohesive ophthalmic viscosurgical device (OVD, Healon GV), a superonasal paracentesis was created. Using an ab interno approach, the preloaded inserter needle was passed through the cornea and the needle was directed across the anterior chamber and implanted in the target quadrant, connecting the subconjunctival space to the anterior chamber. Every effort was made to avoid contact with the corneal endothelium, crystalline lens, and iris during the implantation procedure. If the implant procedure was combined with cataract surgery, successful and uncomplicated cataract surgery was performed prior to the AqueSys XEN implantation. Once the device had been implanted, the anterior chamber was flushed with balanced saline solution to remove viscoelastic fluid followed by hydro sealing of the corneal incision. If cataract surgery was performed, a 10–0 single stitch suture was used for phaco entry.

Patients were instructed to refrain from rubbing their eyes for a few weeks following surgery. BAK-free fluoroquinolone was prescribed QID during the first week postoperative. Prednisolone acetate 1% or equivalent, or difluprednate 0.05% without BAK, was prescribed QID, 1–4 weeks postsurgery, TID, week 5 postoperative, BID, week 6 postoperative, and QD, 7–12 weeks postsurgery.

### 2.7. Statistical Analysis

All statistical analyses were performed using the Analyse-it® software for Microsoft Excel. Continuous variables were checked to meet the normality conditions of Shapiro-Wilk test. Paired and unpaired *t*-tests were used for continuous variables. A Wilcoxon test was employed for all other analyses. Statistical significance was set at *p* < 0.05.

## 3. Results

Twelve eyes of 11 patients with uncontrolled POAG were included in the analysis. The clinical and demographic baseline characteristics are summarized in Table 1. Ten eyes underwent Xen Gel Stent implantation as solo procedure while 2 eyes underwent Xen Gel Stent implantation combined with cataract surgery.

### 3.1. IOP and Medication Use

**Figure 1:**
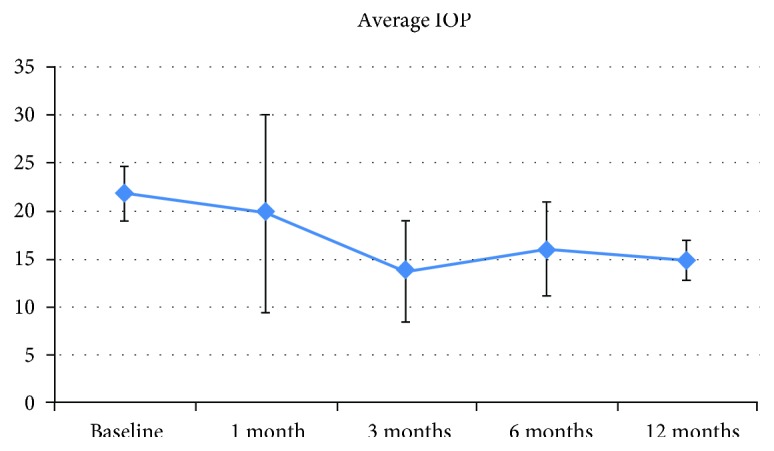
Mean IOP through 12 months postoperative.

**Figure 2:**
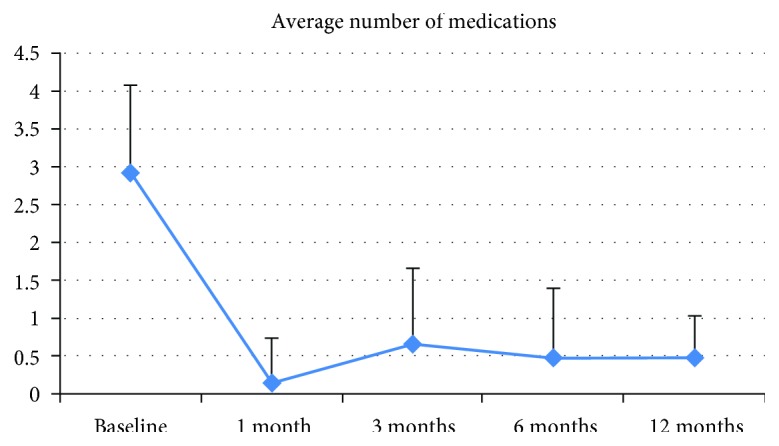
Mean number of antiglaucoma medications through 12 months postoperatively.

At six months postoperative, surgery was a complete success for 8/12 eyes (66.67%), a qualified success for 3/12 eyes (25%), and a failure for 1/12 eyes (8.33%). At 12 months postoperative, five of 10 eyes available for follow-up were in the complete success group; the others were a qualified success requiring one medication. Overall, a 31.62% reduction in IOP and 82.88% in medication versus baseline was achieved (both *p* < 0.001) (Figures 1 and 2; Table 2).

Six eyes required needling: 3 at 1 month and 3 at 6 months (4 “qualified success” and 2 “complete success”). One patient was lost to follow-up at the seven-month visit, and one patient underwent trabeculectomy due to poor IOP control nine months after stent implantation.

### 3.2. Biomicroscopy

Mean bleb area ranged between 2.25 ± 0.45 (1 week) and 2.9 ± 1.37 (1 year) clock hours. Mean height of the bleb (range 0–3) ranged between 1.67 ± 0.49 (1 week) and 1.83 ± 0.58 (1 year). Borders appeared diffused in all patients at all follow-up examinations. Bleb vascularization (scale 0–4 according to a modified Moorfields Bleb grading) was reduced significantly from 1 week (1.58 ± 0.67) to 1 year postoperative (1.1 ± 0.32) (*p* = 0.04).No significant differences between the success and the failure groups at any visit were recorded.

### 3.3. IVCM and AS-OCT

Microcysts at the superonasal conjunctiva detected by IVCM before surgery were scattered, with an optically clear structure, round or oval-shaped surrounded by a hyperreflective wall with no signs of clustering. Postoperatively, microcysts were of different shapes and sizes and were clustered and surrounded by a low reflective wall (Figure 1).

In the postoperative period, mean microcysts area and density increased significantly, whereas the subepithelial connective tissue density decreased significantly till the 6th month and then increased again till the end of the follow-up period. At one year postoperative, the area and density of microcysts at the superonasal conjunctiva were different as compared to the baseline (Table 3, *p* < 0.05 and *p* = 0.05, resp.).

Comparing the “success” and “failure” groups (Table 4), the area and density of epithelial microcysts were higher in success group but without reaching statistical significance (*p* > 0.05). At 12 months postoperative, stromal density was significantly lower in the success group (*p* < 0.05).

**Figure 3:**
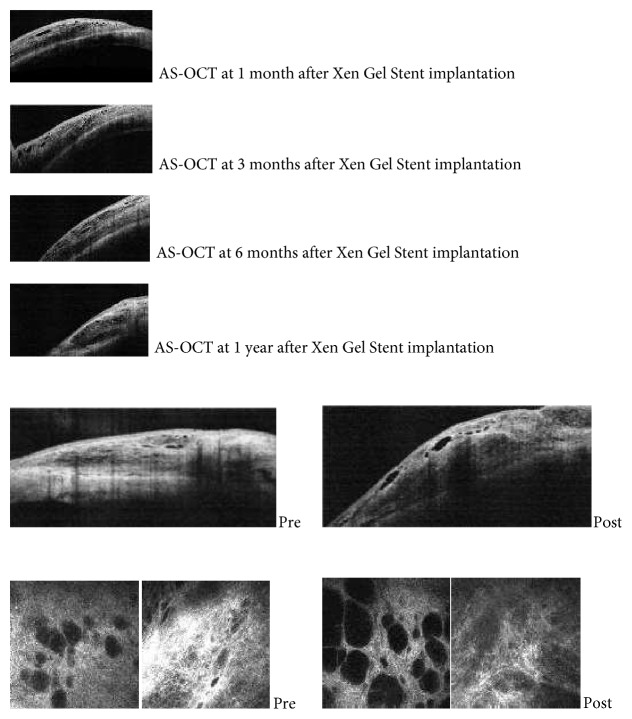
Pre- and postoperative AS-OCT scans.

AS-OCT scans at different follow-up times are shown in Figure 3. There were no significant changes in any of the variables assessed using AS-OCT through the whole postoperative period (*p* > 0.05 for all parameters) (Table 5). Comparing the success and failure groups (Table 6), the maximal height of the bleb and total area of the cystic hypoechoic spaces were significantly higher, whereas bleb wall reflectivity was significantly lower (*p* < 0.05) in functioning versus nonfunctioning blebs at six months postoperative. At one year postoperative, bleb wall reflectivity was significantly different between the two groups (*p* < 0.05), that is, higher in the failure group.

### 3.4. Safety

There was no statistically significant difference in Humphrey visual field testing or BCVA at 12 months postoperative versus baseline (*p* > 0.05) (Table 7). There were also no significant changes in endothelial cell density over one year postoperative (*p* > 0.05); however, cell hexagonality increased significantly (*p* = 0.031). No other safety issues related to the procedure or to the study device were observed.

## 4. Discussion

The XEN Gel Stent is a minimally invasive device designed to offer the same benefits as traditional, gold standard filtering surgeries. A recently published paper reported an IOP reduction around 30% with a reduction of 2.9 glaucoma medications per patient at one year [12]. A previous study with a longer follow-up shows that implantation of the device was associated with a 40% reduction in IOP at 36 months and a 74% reduction in antiglaucomatous medications [8]. In our series, we observed a 31.62% reduction in IOP and an 82.88% reduction in medications use. All patients achieved an IOP < 18 mmHg at 12 months postoperative; five of 10 evaluable eyes required one medication to maintain IOP.

The primary aim of our case series was to examine bleb morphology by means of a variety of imaging techniques, as previously done following trabeculectomy. A slit lamp examination is frequently used to assess bleb morphology; however, in some cases, there is no correlation between the bleb appearance and the IOP, leading to difficulties in assessing the filtering ability and the signs of failure (i.e., scarring processes). Consequently, ophthalmologists are increasingly turning to advanced imaging technologies to gain a clearer understanding of bleb morphology and, in turn, help them differentiate between a functioning and a failing/failed bleb.

Biomicroscopy evaluation of the Xen Gel Stent bleb can be difficult due to the smaller dimensions of the bleb as compared to the blebs typically observed after trabeculectomy. In vivo confocal microscopy showed that, overall, there were significant changes within the conjunctiva over 12 months postoperative. On the other hand, no significant changes in any variables assessed using AS-OCT were found; this can be eventually explained by the absence of a baseline AS-OCT analysis at the intended site of implantation.

Ciancaglini and colleagues proposed a microscopic and macroscopic bleb analyses by undertaking a clinical, IVCM, and AS-OCT assessments of filtering blebs after trabeculectomy [13]. In the present study, we performed both IVCM and AS-OCT analyses of the Xen Gel Stent bleb to detect morphologic features related to stent efficacy. We investigated tissue rearrangement and anatomical modifications induced by stent implantation as well as early signs of the subconjunctival wound healing process responsible of surgery failure.

In this study, IVCM showed significant changes within the conjunctival stroma in all patients one year after surgery. Microcysts within the bleb wall epithelium, firstly detected by Labbè et al. [14] following successful trabeculectomy, were significantly increased in density and area at the 6 months of follow-up visit, suggesting a progressive aqueous percolation after stent implantation. Additionally, at 6 months, stromal reflectivity was significantly lower in the whole superior bulbar conjunctiva with respect to previous observations, suggesting a slower manifestation of tissue rearrangement in deeper layers.

Comparing successful and failed blebs at 12 months, the stromal density was significantly lower in the success group. Increased microcysts and loosely arranged connective tissue/low stromal reflectivity are suggestive of new or increased alternative aqueous humor outflow induced by the stent implantation. Our data show that conjunctival microcysts existed prior to stent implantation. Different hypotheses on epithelial microcysts have been drawn; they are supposed to be hallmarks of transcleral and transconjunctival aqueous percolation or the result of an epithelial disruption, in particular a degeneration at goblet cells level [15].

Ciancaglini et al. [13] previously reported that all IVCM parameters correlated well with the bleb functionality following trabeculectomy whereas, among the AS-OCT parameters, only the bleb wall reflectivity was significantly related to filtering capability. Similarly, in the present study, AS-OCT showed that one year after surgery, there were no significant differences between the success and failure groups except for bleb wall reflectivity, which was higher in nonfunctioning blebs. An early study by Addicks et al. [10] found that encapsulated blebs (where the bleb wall was composed of dense collagenous connective tissue) had high reflectivity, while Leung and colleagues noted low–medium reflectivity in the OCT images of functioning blebs [16].

Considering the AS-OCT cross-sections, neither biometric parameters nor bleb wall reflectivity changed over time, suggesting that the bleb probably acquires its definitive macroscopic appearance by the first month after stent implantation. At the third and sixth months postoperative, the maximal height of the bleb and the total area of cystic hypoechoic spaces were significantly higher in the success group probably due to a higher amount of fluids in filtering blebs. Bleb stromal reflectivity was significantly different between the two groups at all follow-up visits. We hypothesize that the density of the bleb connective tissue may be the determining factor for the long-term functionality of the bleb.

Longitudinal scans, AS-OCT, proved to be useful in checking the correct placement of the device and were useful in the present study to evaluate consistent areas of the bleb over the follow-up.

Additionally, AS-OCT, as compared to biomicroscopy and IVCM, provided “intrableb imaging” and a macroarchitectural analysis of the bleb as a whole.

This study presents some limitations. It has a small sample size, has a relatively short follow-up, and lacks an early postoperative evaluation to more stringently monitor the bleb development. A more standardized approach in bleb evaluation with imaging techniques is also warranted.

The presence of inflammatory cells, indicators of inflammation, and subsequent fibrosis in the conjunctival epithelium was not methodically investigated; however, it could provide a valuable predictive tool and deserves further investigations. It is also possible that AS-OCT may misrepresent bleb structure and artifacts [17]. Finally, the sample size is small and the follow-up is quite short; larger scale studies with longer follow-up are required.

To the best of our knowledge, this is the first study to evaluate bleb morphology following implantation of the XEN Gel Stent. We showed the wound healing may proceed, but by using biomicroscopy, the bleb may not appear to change, resulting in aqueous outflow decrease and an increase in IOP. Consequently, we propose a combined clinical and instrumental approach that could enable the clinician to functionally evaluate the XEN Gel Stent filtering bleb in the postsurgical follow-up.

Our findings also show that there were statistically significant reductions in both IOP and medication use following implantation of the XEN Gel Stent in this group of POAG patients, and no safety issues related to the procedure or to the study device were observed. Overall, implantation of the XEN Gel Stent appears to be an effective and safe procedure for the treatment of POAG.

## Figures and Tables

**Table 1:** Patient demographics and baseline characteristics.

Patients	11
Eyes	12
Gender (M/F)	5/6
Age (years)	71.3 ± 10.0
CCT (*μ*m)	548.7 ± 44.9
Snellen BCVA	6.38 ± 3.23/10
MD (dB)	−12.5 ± 8.69 dB
Preoperative IOP (mmHg)	21.79 ± 2.8 mmHg
Number of antiglaucoma medications at baseline	2.92 ± 1.16

BCVA: best corrected visual acuity; CCT: central corneal thickness; MD: mean deviation of visual field test.

**Table 2:** IOP and number of medications over one year postoperatively.

Baseline		1 month		3 months		6 months		1 year	
IOP	Number of medications	IOP	Number of medications	IOP	Number of medications	IOP	Number of medications	IOP	Number of medications
21.8 ± 2.8	2.92 ± 1.16	19.8 ± 10.3	19.8 ± 10.3 0.17 ± 0.58	13.8 ± 5.2	0.67 ± 0.98	16.0 ± 4.8	0.50 ± 0.90	14.9 ± 2.1	0.50 ± 0.53

**Table 3:** In vivo confocal microscopy data preoperatively and after XEN Gel Stent implantation.

	MMA	MMD	SCTD
Preop	773.3 ± 1361.6	7.64 ± 15.12	91.25 ± 23.20
Month 3	15564.9 ± 12264.1	56.75 ± 44.90	83.85 ± 14.90
Month 6	16255.1 ± 14056.7	68.03 ± 37.08	70.30 ± 20.71
Month 12	12159.4 ± 16634.5	77.87 ± 95.63	96.55 ± 32.38
*p* value preop versus month 6	0.002	< 0.001	0.037
*p* value preop versus month 12	0.059	0.003	0.679

MMA: mean microcysts area; MMD: mean microcysts density; SCTD: subepithelial connective tissue density. Data are expressed as mean ± standard deviation. For the analysis of variables, the Student *t*-test was applied. *p* values <0.05 were considered statistically significant.

**Table 4:** In vivo confocal microscopy data: success versus failure groups.

	MMA		MMD		SCTD	
	Month 6	Month 12	Month 6	Month 12	Month 6	Month 12
Success group	19,407 ± 15,796	12,502 ± 19,633	50.12 ± 30.16	86.47 ± 91.47	62.12 ± 18.02	72.69 ± 14.53
Failure group	13,665 ± 8120	11,816 ± 15,389	44.17 ± 8.42	69.27 ± 109.66	86.64 ± 16.75	120.4 ± 26.91
*p* value success versus failure	0.20	0.95	0.35	0.79	0.05	0.01

MMA: mean microcysts area; MMD: mean microcysts density; SCTD: subepithelial connective tissue density. Data are expressed as mean ± standard deviation. For the analysis of variables, the Student *t*-test was applied. *p* values < 0.05 were considered statistically significant.

**Table 5:** Anterior segment OCT data over 1 year postoperatively.

	Maximal height of the bleb	Total area of the hyporeflective cystic spaces	Bleb wall reflectivity	Bleb wall thickness
Month 1	492.4 ± 281.8	802.1 ± 96.0	136.6 ± 49.7	206.3 ± 228.8
Month 6	586.0 ± 418.7	828.8 ± 991.0	129.6 ± 34.2	102.7 ± 36.6
Month 12	462.4 ± 328.1	555.0 ± 572.6	130.7 ± 55.7	158.8 ± 76.3
*p* value (month 1 versus 12)	NS	NS	NS	NS

Data are expressed as mean ± standard deviation. For the analysis of variables, the Student *t*-test was applied. *p* values < 0.05 were considered statistically significant. NS: nonsignificant.

**Table 6:** Anterior segment OCT data: success versus failure groups.

	Maximal height of the bleb				Total area of the hyporeflective cystic spaces				Bleb wall reflectivity				Bleb wall thickness			
	1st month	3rd month	6th month	12th month	1st month	3rd month	6th month	12th month	1st month	3rd month	6th month	12th month	1st month	3rd month	6th month	12th month
Success group	672.6 ± 344.3	662.8 ± 361.7	841.8 ± 347.3	570.1 ± 326.30	1616.8 ± 875	937.6 ± 787.7	1123.8 ± 806	588 ± 344.4	105.6 ± 32.5	121.8 ± 37.4	102.2 ± 20.9	85.3 ± 11.9	280 ± 321	99.6 ± 36.5	120.4 ± 21.5	146.8 ± 51.7
Failure group	329.5 ± 70	238 ± 60	280 ± 149.9	419.4 ± 374.4	198.5 ± 156.3	161.3 ± 111.9	127 ± 120.2	647.5 ± 811.8	194.5 ± 29.0	190.3 ± 12	165.6 ± 47.3	167.0 ± 48.8	156 ± 19.8	82 ± 42.4	96 ± 62.2	168.5 ± 96.8
*p* value	0.016	0.005	0.002		0.028	0.008	0.004		<0.001							
				0.414				0.880		<0.001	0.042	0.018	0.629	0.555	0.79	0.681

Data are expressed as mean ± standard deviation. For the analysis of variables, the Student *t*-test was applied. *p* values < 0.05 were considered statistically significant.

**Table 7:** Safety parameters.

	BCVA	MD	CD	SD	CV	6A
Baseline	6.38 ± 3.23	−12.5 ± 8.69	2038.3 ± 724.3	226.1 ± 92.8	40.0 ± 5.4	54.56 ± 8.93
1st week	6.29 ± 2.77	—	—	—	—	—
1st month	6.29 ± 2.83	—	2008.89 ± 568.52	209.11 ± 77.78	39 ± 8.46	56.67 ± 9.22
3rd month	6.38 ± 3.08	—	1798.82 ± 686.77	283.36 ± 160.05	41.45 ± 9.23	53.09 ± 7.79
6th month	6.38 ± 3.08	−12.3 ± 8.61	1859.27 ± 732.67	210 ± 111.35	32.36 ± 6.54	61.73 ± 6.17
1 year	7 ± 2.11	−12.47 ± 8.63	2111.56 ± 443.89	167.56 ± 27.46	34.56 ± 4.45	63.22 ± 6.24

BCVA: best corrected visual acuity; CD: cell endothelial density; SD: standard deviation; CV: coefficient of variation; 6A: cell hexagonality; MD: mean deviation at visual field test.
